# Minimizing sevoflurane wastage by sensible use of automated gas control technology in the flow-i workstation: an economic and ecological assessment

**DOI:** 10.1007/s10877-021-00803-z

**Published:** 2022-01-03

**Authors:** Alain F. Kalmar, Nicky Van Der Vekens, Fréderic De Rydt, Silvie Allaert, Marc Van De Velde, Jan Mulier

**Affiliations:** 1 Department of Anesthesiology, Reanimation and Intensive Care, AZ Sint Jan Brugge-Oostende, Brugge, Belgium; 2 Department of Anesthesia, Ghent University, Gent, Belgium; 3 Departmen of Anesthesia and Critical Care Medicine, Maria Middelares Hospital, Gent, Belgium; 4 Department of Cardiovascular Sciences, KULeuven, Leuven, Belgium; 5 Department of Anesthesiology, UZLeuven, Leuven, Belgium; 6 Department of Anesthesiology, KULeuven - University of Leuven, Leuven, Belgium

**Keywords:** Volatile anaesthetics, Pollution, Flow-i, Automated gas control

## Abstract

Both ecological and economic considerations dictate minimising wastage of volatile anaesthetics. To reconcile apparent opposing stakes between ecological/economical concerns and stability of anaesthetic delivery, new workstations feature automated software that continually optimizes the FGF to reliably obtain the requested gas mixture with minimal volatile anaesthetic waste. The aim of this study is to analyse the kinetics and consumption pattern of different approaches of sevoflurane delivery with the same 2% end-tidal goal in all patients. The consumption patterns of sevoflurane of a Flow-i were retrospectively studied in cases with a target end-tidal sevoflurane concentration (Et_sevo_) of 2%. For each setting, 25 cases were included in the analysis. In Automatic Gas Control (AGC) regulation with software version V4.04, a speed setting 6 was observed; in AGC software version V4.07, speed settings 2, 4, 6 and 8 were observed, as well as a group where a minimal FGF was manually pursued and a group with a fixed 2 L/min FGF. In 45 min, an average of 14.5 mL was consumed in the 2L-FGF group, 5.0 mL in the minimal-manual group, 7.1 mL in the AGC4.04 group and 6.3 mL in the AGC4.07 group. Faster speed AGC-settings resulted in higher consumption, from 6.0 mL in speed 2 to 7.3 mL in speed 8. The Et_sevo_ target was acquired fastest in the 2L-FGF group and the Et_sevo_ was more stable in the AGC groups and the 2L-FGF groups. In all AGC groups, the consumption in the first 8 min was significantly higher than in the minimal flow group, but then decreased to a comparable rate. The more recent AGC4.07 algorithm was more efficient than the older AGC4.04 algorithm. This study indicates that the AGC technology permits very significant economic and ecological benefits, combined with excellent stability and convenience, over conventional FGF settings and should be favoured. While manually regulated minimal flow is still slightly more economical compared to the automated algorithm, this comes with a cost of lower precision of the Et_sevo_. Further optimization of the AGC algorithms, particularly in the early wash-in period seems feasible. In AGC mode, lower speed settings result in significantly lower consumption of sevoflurane. Routine clinical practice using what historically is called “low flow anaesthesia” (e.g. 2 L/min FGF) should be abandoned, and all anaesthesia machines should be upgraded as soon as possible with automatic delivery technology to minimize atmospheric pollution with volatile anaesthetics.

## Introduction

Volatile anaesthetics are widely used hypnotics with desirable pharmacological properties. A major drawback, however, is that these gases are eventually discarded into the atmosphere where they contribute significantly to the greenhouse effect. Global emissions of fluorinated volatile anaesthetics in 2014 equaled three million tons of CO_2_, of which eighty percent was from desflurane alone [1]. As the climate emergency becomes ever more apparent, threatening to decimate complete ecosystems and triggering vast medical and societal emergencies [2], it is everyone’s duty to minimize their personal ecological impact. Given the strong heat-trapping potency of volatile anaesthetics, anaesthetists have an important responsibility in this regard [3]. Remarkably simple choices made by the anaesthetist can reduce the climate impact by orders of magnitude without negatively impacting the quality of care. As far as volatile anaesthetics still being desirable, a minimal understanding of their climatic effects dictates that the most important steps should be to avoid desflurane and N_2_O, and to make optimal use of modern technology to minimize fresh gas flow [4]. In addition to environmental benefits, reducing the wasteful use of volatile anaesthetics can provide significant financial savings.

Resulting from the complexities of the atmospheric physics and chemistry, which is extensively described elsewhere, sevoflurane has a global heating effect which is 349 times worse than CO_2,_ while desflurane is even 3714 times worse [3]. Because volatile anaesthetics are widely and often continuously used in operating theatres, the total consumption of volatile anaesthetics in conventional low-flow settings may easily amount to 40 L of sevoflurane or 100 L of desflurane per anesthesia workstation per year. This amounts to a financial cost of well in excess of 16,000€ per year of volatile anaesthetics, and a greenhouse gas equivalent of 21 metric tons of CO_2_ for sevoflurane or 542 metric tons for desflurane [3, 5]. As such, a reduction in volatile anaesthetic waste would lead to significant financial savings—easily covering the additional cost of modern equipment—and a huge reduction in atmospheric pollution. As a reference, one roundtrip intercontinental flight Brussels-New York in economy class results in 2 metric tons of CO_2_ emissions per person.

While technological innovations, like pulse oximetry and continuous gas analysis have made conventional manual minimal flow anaesthesia safe, it still demands expertise and continuous attention [6, 7]. The addition of automated low-flow software finally enables optimized carrier gas flows and volatile agent administration to precisely secure the delivery of the desired gas mixture while effortlessly minimizing waste [8, 9]. The Flow-i anaesthesia machine (Getinge, Goteborg, Sweden), for instance, can be supplied with AGC® (Automated Gas Control). This permits the anaesthetist to set the appropriate speed—on a numeric scale from 1 (slow) to 8 (fast)—to reach the selected end-tidal concentrations of volatile anaesthetics. The AGC® algorithm gradually reduces the FGF to a minimal rate depending on the patient’s oxygen consumption, resulting in environmental and economic advantages. [10, 11] Automated software obviates frequent manual adjustment of the settings during minimal flow anaesthesia and optimizes the stability of the administered anaesthetics and inspiratory oxygen fraction (F_i_O_2_) [12]. Except for rare situations, such as carbon monoxide poisoning, there are no contraindications to perform minimal flow anaesthesia [6]. Also the increased cost due to elevated CO_2_ absorbent consumption at minimal flow does not outweigh the volatile anaesthetics economised [13]. The current study aims to compare the rate of sevoflurane consumption in conventional low flow anaesthesia (2 L/min FGF), manually adjusted minimal gas flow and software versions AGC_4.04_ and AGC_4.07_.

## Methods

After institutional ethical approval (MMS.2021.004), the data of the digital charting system (ICCA, Philips, Amsterdam, Netherlands) were analysed. These records include all intraoperative data at 15 s interval, in addition to any anaesthetic intervention such as provided airway and administered drugs. All Flow-i workstations were equipped with either AGC® version V4.04.01 or version V4.07.00. The cumulative amount of sevoflurane consumption reported by the Flow-i is automatically recorded with a precision of 0.1 mL.

Data from all cases after 01/10/2019 were evaluated and the first 25 subsequent cases in each of the following groups meeting the inclusion criteria were extracted and analyzed: AGC® version V4.04.01 in speed 6; AGC® v4.07.00 in speed 2, 4, 6 and 8; a fixed 2 L/min fresh gas flow (2LFGF); or a manual adjustment (MGF) to the lowest possible flow of 300 mL/min. In both AGC software versions the FGF was automatically reduced to a minimal rate of 300 mL/min. In MGF, a few seconds of intermittently higher flows were often applied to attain or maintain the desired end-tidal sevoflurane concentration (Et_Sevo_). In all cases, an F_i_O_2_ of 80%, and a target Et_Sevo_ of 2% was pursued. The primary outcome variable of interest was the cumulative consumption of sevoflurane after 45 min.

### Data registration and analysis

All anaesthetic data were extracted and subsequently imported into Microsoft Excel 2010® (Microsoft, Redmond, USA) for analysis. Assuming a normal distribution of the consumption data, we considered a mean difference of 1 mL after 45 min between AGC and minimal flow to be relevant (estimated SD of 1.1 mL, based on pilot data). To detect this difference with an α-error of 0.05 and a power of 0.95, a total of 25 records was needed in each group [14].

Normality was tested with the Kolmogorov–Smirnov test. Continuous data are expressed as mean (SD). For statistical analysis and visualization, the individual records were synchronized at the moment (T0) after initiation of ventilation that Et_Sevo_ exceeded 0.2%. Recordings with at least 55 min sevoflurane administration were included in the analysis.

**Fig. 1:**
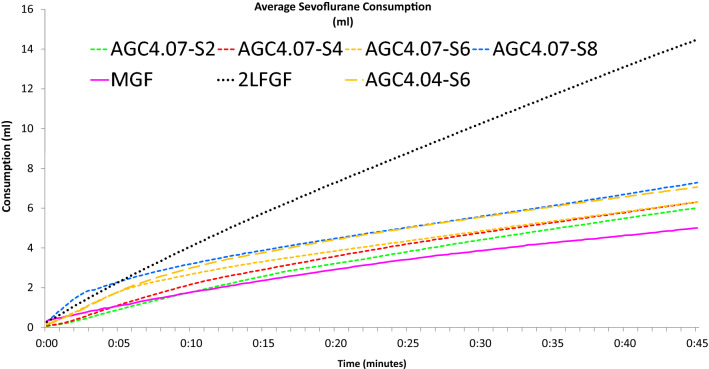
Average cumulative sevoflurane consumption in different modes of sevoflurane administration in the flow-i ventilator. AGC speed 6 with the AGC4.04 algorithm, AGC speed 2, 4, 6, 8 with the newer AGC4.07 algorithm, manual minimal gas flow (MGC), and constant 2 L/min FGF (2L FGF)

**Fig. 2:**
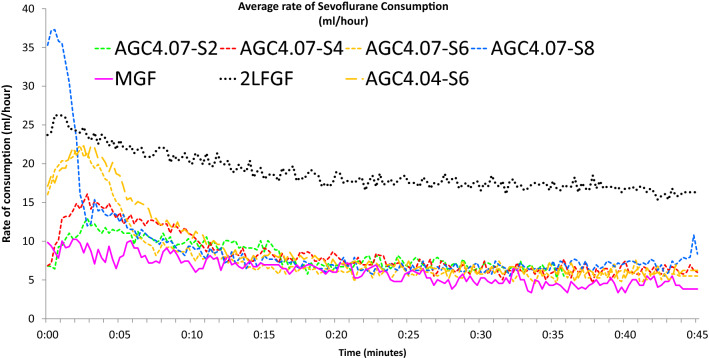
Average rate of sevoflurane consumption in different modes of sevoflurane administration in the flow-i ventilator. AGC speed 6 with the AGC4.04 algorithm, AGC speed 2, 4, 6, 8 with the newer AGC4.07 algorithm, manual minimal gas flow (MGC), and constant 2 L/min FGF (2L FGF)

**Fig. 3:**
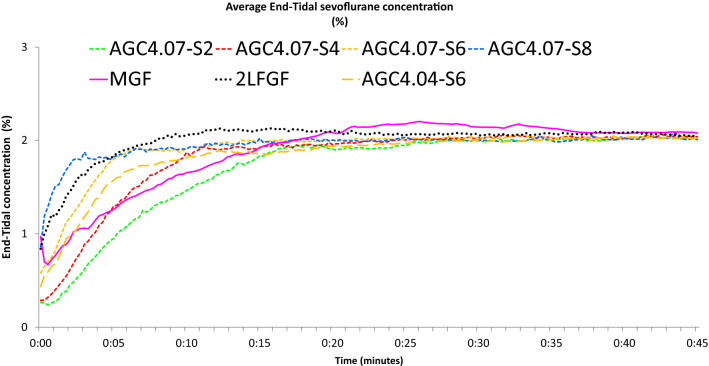
Average end-tidal sevoflurane concentration in different modes of sevoflurane administration in the flow-i ventilator. AGC speed 6 with the AGC4.04 algorithm, AGC speed 2, 4, 6, 8 with the newer AGC4.07 algorithm, manual minimal gas flow (MGC), and constant 2 L/min FGF (2L FGF)

**Fig. 4:**
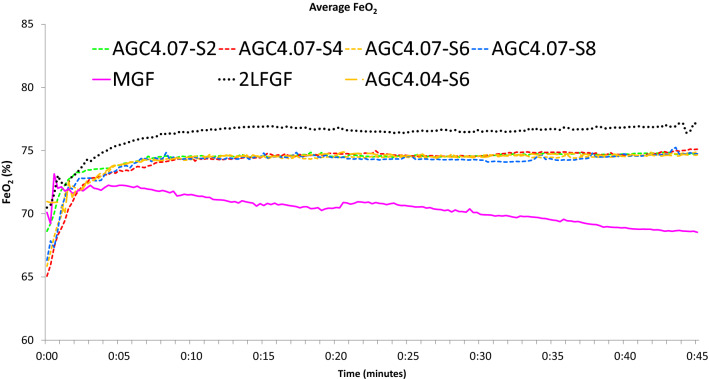
Average end-tidal O2 concentration in different modes of sevoflurane administration in the flow-i ventilator. AGC speed 6 with the AGC4.04 algorithm, AGC speed 2, 4, 6, 8 with the newer AGC4.07 algorithm, manual minimal gas flow (MGC), and constant 2 L/min FGF (2L FGF)

**Fig. 5:**
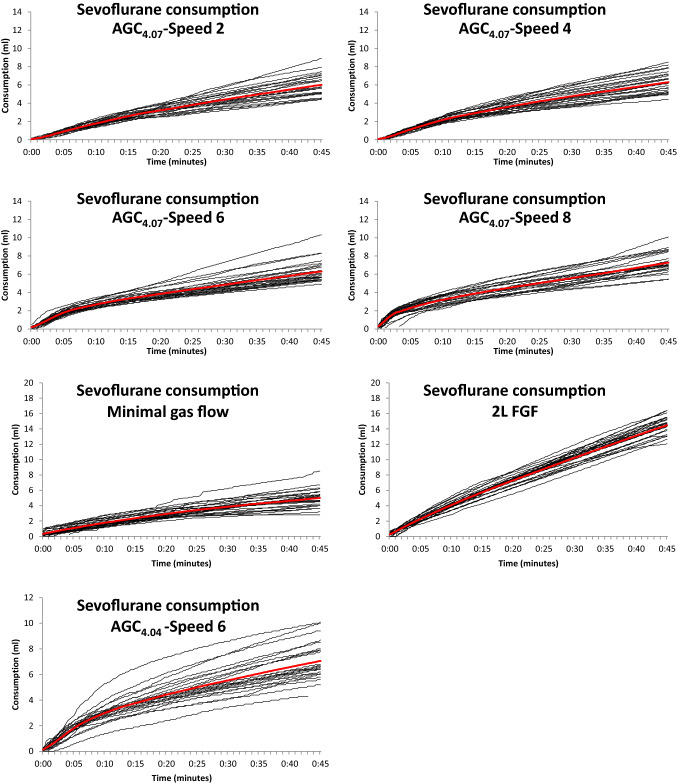
Individual (thin lines) and Average (thick red line) cumulative sevoflurane consumption in different modes of sevoflurane administration in the flow-i ventilator. AGC speed 6 with the AGC4.04 algorithm, AGC speed 2, 4, 6, 8 with the newer AGC4.07 algorithm, manual minimal gas flow (MGC), and constant 2 L/min FGF (2L FGF)

**Fig. 6:**
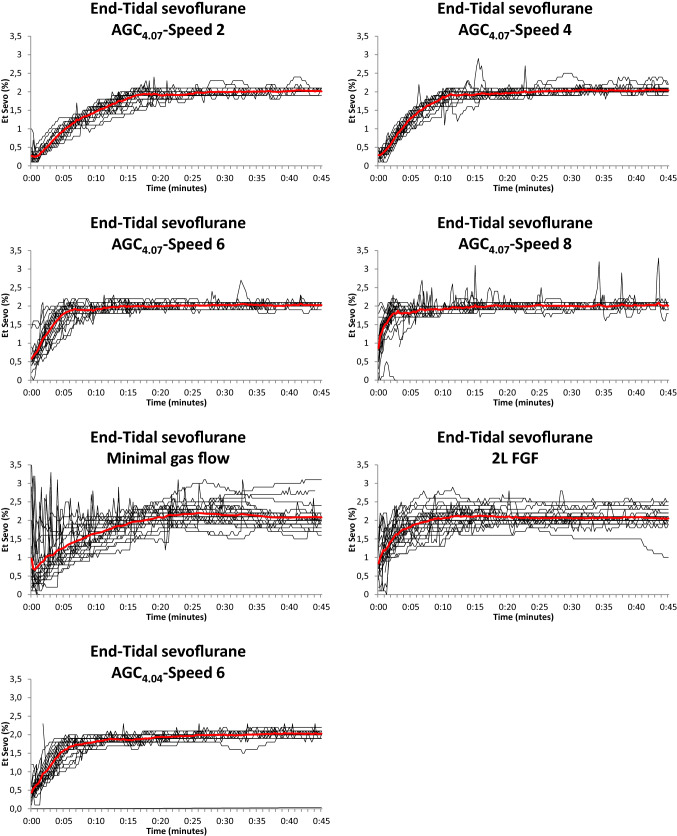
Individual (thin lines) and Average (thick red line) end-tidal sevoflurane concentration in different modes of sevoflurane administration in the flow-i ventilator. AGC speed 6 with the AGC4.04 algorithm, AGC speed 2, 4, 6, 8 with the newer AGC4.07 algorithm, manual minimal gas flow (MGC), and constant 2 L/min FGF (2L FGF)

For comprehensive comparison of the different groups, the average values were shown in Figs. 1, 2, 3, 4. For visual assessment, the evolution of the individual curves were depicted in Figs. 5, 6.

The rate of sevoflurane consumption at a certain minute (R_m_, expressed as mL/hour) was calculated as the increase in cumulative consumption over the coming minute: R_m_ = (C_(m+1)_ − C_m_)*60.

The average (SD) values of the analysed variables were determined at 5, 15, 30 and 45 min. ANOVA followed by an Unpaired T-test was used to determine differences between groups. Significance was set at P < 0.05.

## Results

Patient characteristics and cumulative consumption of each group at 5, 15, 30 and 45 min are shown in Table 1.

**Table 1:** Patient characteristics and cumulative consumption:

	AGC_4.07_ Speed 2	AGC_4.07_ Speed 4	AGC_4.07_ Speed 6	AGC_4.07_ Speed 8	2LFGF	AGC_4.04_ Speed 6	MGF	P value
Age	51 (16)	53 (18)	52 (15)	53 (15)	59 (15)	55 (18)	58 (14)	0.444
Weight	80 (22)	75 (18)	75 (22)	83 (22)	72 (12)	82 (21)	74 (18)	0.310
Gender (M/F)	14/11	12/13	16/9	10/15	14/11	11/14	16/9	
Cumulative consumption								
At 5 min	0.9 (0.2)*	1.1 (0.2)*	1.8 (0.3)*	2.3 (0.4)	2.3 (0.3)*	1.8 (0.4)*	1.1 (0.4)	< 0.001
At 15 min	2.6 (0.3)*	2.9 (0.4)*	3.3 (0.4)*	3.9 (0.6)*	5.8 (0.6)*	3.8 (0.9)*	2.4 (0.5)	< 0.001
At 30 min	4.4 (0.7)	4.8 (0.8)	4.9 (0.8)*	5.6 (0.8)*	10.2 (0.8)*	5.5 (1.2)*	3.9 (0.8)	< 0.001
At 45 min	6.0 (1.2)	6.3 (1.1)	6.3 (1.3)*	7.3 (1.1)*	14.5 (1.2)*	7.1 (1.4)*	5.0 (1.2)	< 0.001

Average(SD) Age, Weight and cumulative sevoflurane consumption, gender distribution (Male/Female) in the different groups. Twenty-five patients were included in each group

*Significant difference between adjacent columns

Figures 1, 2, 3 and 4 show the cumulative consumption of sevoflurane (mL), the rate of sevoflurane consumption (mL/hour), the average Et_sevo_ (%) and the expiratory O_2_ concentration (%) in each group, respectively. Figures 5 and 6 show the individual patient recordings (thin lines), as well as average values (thick lines) of cumulative consumption and of Et_sevo_ respectively in each group.

In the 2L-FGF group, the rate of sevoflurane consumption remains high during the entire 45 min, whereas in the AGC groups there is a significant drop after three minutes (Fig. 2). In all groups, except the 2L-FGF group, although initial consumption rates vary significantly, after 10 min the rate of consumption becomes comparable. (Fig. 2).

Sevoflurane consumption in speed 6 with the new algorithm—AGC_4.07_—initially had an equal consumption compared to the old algorithm—AGC_4.04_—but thereafter, the newest algorithm spent significantly (P = 0.027) less sevoflurane to maintain its target. The consumption in the most economical AGC-speed 2 was still significantly (P = 0.004) higher than in manual minimal flow (Fig. 1, Table 1).

## Discussion

Scientists have a moral obligation to clearly warn humanity of any catastrophic threat. On this basis, supported by overwhelming evidence, Scientific American declared we are living in a climate emergency [15]. As the adverse effects of climate change are much more severe than expected and now threaten both the biosphere and humanity, every effort must be made to reduce emissions of greenhouse gasses. While this call is resonating increasingly loudly, many anaesthesiologists are insufficiently aware of the extent to which their daily choices have an impact thereon, and how minimal adjustments in daily practice can dramatically reduce the environmental impact of anaesthesia without compromising anaesthetic end-tidal concentration corresponding to anaesthetic depth.

Following initial clinical administration, volatile anaesthetics can be reused after their passage through the carbon dioxide absorber. When low fresh gas flow is applied, less gas must be vented to the exhaust system and consequently less sevoflurane must be added into the breathing system. An increased consumption of CO_2_ adsorbents is seen but does not have a global negative impact on the financial price tag [13]. With the modified formulations in the current CO_2_ absorbents in the last decades, compound A formation and toxicity in humans at low flows is no longer a concern [16, 17] and apart from rare conditions such as CO intoxication, there is no reason to avoid minimal gas flow [6]. Still, at lower FGF, there will be an increased consumption of absorbents, with consequently also the pollution associated with their production and destruction. Even though neither the plastic package nor the soda lime are ecotoxic when landfilled, an amount of CO_2_ is released during production and incineration.

A large sodalime canister contains 1200 g sodalime and 200 g plastic, the production and incineration of which results in CO_2_ emissions of around 1.3 kg, corresponding with 2.4 mL of sevoflurane [5, 18, 19]. As such, it is striking that the pollution owing to even only the excess sevoflurane consumption when using 2 L/min FGF instead of AGC-speed 2 in the first 12 min of anesthesia in only one average patient equals the pollution attributable to the production and incineration of a large sodalime canister.

Our results confirm that cumulative volatile anaesthetic consumption—and therefore pollution—can be significantly reduced by using AGC® or by manually applying minimal FGF, compared even to the traditionally called “low flow” (2 L/min FGF) anaesthesia. Cumulative sevoflurane consumption after 45 min in the Minimal group was still 17% lower compared to the least consuming AGC® (5.00 mL versus 6.02 mL). When comparing different speed settings in AGC® V4.07.00, consumption in speed 8 (fastest) was 21% higher than in speed 2 (7.28 vs 6.02 mL). It takes about 15 min in speed 2 to reach Et_sevo_ of 2% ± 0.2% compared to 3 min in speed 8. While it is often important to quickly reach this target concentration, in most clinical cases, a period of minimal patient stimulation occurs after intubation while the induction dose of propofol still provides a strong hypnotic effect. As such, swiftly attaining an Et_sevo_ of 2% would in most cases result in an unnecessarily high dose of hypnotics often with adverse haemodynamic effects, in addition to needless waste and pollution.

Figure 2 shows that the rate of sevoflurane consumption continuously remains highest in the 2L-FGF group, whereas there is a significant drop after the first minutes in all other groups. In all groups, except the 2L-FGF group, although initial consumption rates vary significantly, the rate of consumption becomes similar after 10 min.

Still, a slightly lower consumption in the MGF group compared to all AGC groups persists. Figure 4 shows that in AGC, the algorithm manages to stabilize the expiratory O_2_ (FeO_2_), while in MGF, FeO_2_ slowly decreased. The higher FGF required to stabilize FeO_2_ probably resulted in somewhat higher consumption of sevoflurane. This observation suggests that a lower target FiO_2_ when using AGC will result in lower consumption of volatile anesthetics.

The rate of sevoflurane consumption in speed 6 in both AGC V4.07.00 and V4.04.01 was similar after 5 min (1.8 mL in both groups), but was 12% lower after 45 min in the newest software version V4.07.00 (6.3 mL vs 7.1 mL) (Fig. 2, Table 1).

Comparison with reports on the first AGC software version shows a steady trend of continuous improvement. Carette et al. reported for version 4.0.0 at speeds 2, 4 and 6 a cumulative consumption after 30 min of 5.0 mL, 6.1 mL and 7.0 mL sevoflurane, respectively [9]. Our results for the same speed settings show a consumption of 4.4 mL, 4.8 mL, and 4.9 mL after 30 min. This emphasizes that even an update to the most recent software version easily results in an annual saving of 2500 mL of sevoflurane and an equivalent of 1326 kg of CO_2_ in emissions. Likewise, since even conventional “low flow” anaesthesia at 2 L/min FGF results in a consumption of 240% compared to AGC speed 2, institutions lacking automated gas delivery technology should be encouraged to invest in more modern equipment. Simply replacing routine 2 L/min FGF by AGC, at 250 working days/year, 8 h/day, implementation of AGC would result in annual savings of 17,000 mL of sevoflurane for each machine, equalling 9 metric tons of CO_2_ and costing circa 6000€. Since the AGC software costs approximately 5000€, this investment would be paid back in less than a year. If completely new workstations are required, a purchase price, including the most advanced software of, generously estimated, 45,000€ is recovered in less than 8 years. If a higher FGF than 2 L/min is often applied, the purchase price is obviously recovered much faster.

Analogously, proper investment in training and raising awareness of the anaesthesiologists to make maximal and conscious use of this new technology would be highly beneficial to maximise the economic and ecologic benefits.

On a societal level, it is appropriate to consider the social cost of CO_2_ as well. Since anthropogenic climate change will cause excess mortality due to heat stress, this mortality cost is estimated at 37$ to 258$ per ton of emitted CO_2_ equivalents, depending on model assumptions [20]. This difference in societal cost resulting from climate change between 2L/min FGF and AGC would thus amount to annually between 337$ and 2353$ when using sevoflurane and between 8322$ and 58,400$ when using desflurane per workstation [3, 20].

Figure 6 shows that, while practicing manual minimal flow anaesthesia with the Flow-i is reliable, the stability of Et_sevo_ when using AGC®-technology is significantly better without the need of any adjustments of the vaporizer or fresh gas flow settings. On top of an increased convenience for the anaesthetist, AGC improved stability and arguably additionally improves safety, and should therefore be advocated also from a clinical perspective. As such, while manual minimal flow yields lower consumption in the first few minutes, we regard this primarily a directional message to the software developers, but as a clinical recommendation we would encourage systematically using AGC mode.

Compared to other studies, focused on the pharmacokinetics, it is noteworthy that the speed to reach 90% of target in our findings is slightly faster than in software version 4.0.0. Carette et al. showed that 90% of target was reached in speed 2, 4 and 6 after 15, 10 and 6 min, respectively. In our observations in version 4.07.00 we reached 90% of target with the same speed settings after 13:45, 09:30 and 05:00 min:sec, respectively [9]. Likewise, De Medts et al. reported that when using desflurane, 90% of target was reached after 16:00, 10:45 and 06:45 min:sec [21]. Remarkably, in a study using the zeus workstation, De Cooman et al. reported that the use of automated closed-circuit anesthesia (the equivalent of AGC in flow-i) resulted in significantly higher consumption than with manual minimal flow despite that in manual setting they maintained the initial FGF of 6 L/min for 3 min, followed by a continuous FGF of 0.7 L/min. They attributed this to a high initial FGF, and intermittent flushing to reduce N_2_ accumulation. Our study, in contrast, suggests that AGC in flow-i is actually able to more economically achieve the desired target, although a comparison is difficult since in their study N_2_O was associated with the volatile anesthetics [22].

The most important limitation of this study, is its retrospective nature. While in all patients an end-tidal concentration of 2% was pursued, some bias that might affect the results cannot be excluded. Nevertheless, analysis of the individual curves (Fig. 5 and 6) suggests reliable consumption rates and analysis of the patient characteristics (Table 1) indicates comparable patients in each group. Secondly, at the moment of the data recordings, the software was set to a lowest FGF in AGC mode of 0.3 L/min, while it also permits presetting a lower limit of 0.1 L/min, which would likely further improve the economics of AGC. Thirdly, by institutions protocol, an FiO_2_ of 80% was always used in AGC. The recent consensus recommendations, however, prescribe an FiO2 of ≥ 40%, which may reduce the fresh gas flow, thereby decreasing the consumption in the first minutes in AGC [23]. The 16% higher sevoflurane consumption we still observed in the most economical AGC setting compared to manual minimal flow (6.0 vs 5.0 mL after 45 min) might therefore diminish after adjustment of the minimal FGF setting to 0.1 L/min and targeting a lower FiO_2_, although the difference in consumption mainly occurred during the initial minutes when the FGF was much still higher than 0.3 L/min. Still, our results demonstrate that future software upgrades may yield further improvements. Fourth, to enable correct comparison between groups, this analysis was limited to cases where the target concentration was set after induction of anaesthesia and not adjusted thereafter. We may expect that frequent changes of the target concentration during the procedure will have a varying influence on the consumption figures in the different AGC settings. Fifth, regarding the calculations on greenhouse gas release, the ultimate impact is approximately 10% worse, since the waste emissions during industrial manufacturing of the sevoflurane releases roughly 10% of the CO_2_ equivalents that are released during use, depending on the production methods [24]. Finally, the potential reduction in N_2_O emissions was not investigated in this study, because the use of N_2_O in the hospital was already phased out years ago for ecological reasons.

## Conclusion

Implementation of AGC technology results in significant economic and ecological savings. Even compared to conventional “low flow anaesthesia” of 2 L/min FGF, AGC is much more efficient. The excellent stability of AGC requiring minimal operator interventions represents a major advantage for AGC in terms of waste reduction, workload and patient safety. Implementation of automatic gas control technology permits safe and convenient reduction of anaesthetic waste of easily 50%; this technology would therefore result for each machine in an annual financial saving well over 5000–10,000€ and an equivalent of 11 or 270 tons of CO_2_ emission when using sevoflurane or desflurane, respectively. The financial savings resulting from the implementation of AGC in most cases suffices comfortably to finance the adoption of advanced anesthesia workstations. These financial considerations may vary depending on the region, because different business models and processes may be used for who is paying for medication versus the technical equipment. It is, however, abundantly clear that on a hospital or society level the investment in automated systems generously pays off. On an ecological level, it should be emphasized that the patient receives the same level of anaesthesia, with even increased safety, but with a lower cost for both society and the biosphere.

Our results also demonstrate that there may still be room for significant improvement of the AGC algorithms to match the excellent stability with a further improved efficiency, particularly in the early wash-in period. Our results show that (even) what historically is called “low flow anaesthesia” should be abandoned, and all anesthesia workstations should be upgraded as soon as possible in order to benefit from automated gas delivery.

## Data Availability

All data is visible in the figures. The spreadsheet with numerical measurement values is available upon request.
