# Correction to: Minimizing sevoflurane wastage by sensible use of automated gas control technology in the flow‑i workstation: an economic and ecological assessment

**DOI:** 10.1007/s10877-022-00832-2

**Published:** 2022-02-14

**Authors:** Alain F. Kalmar, Nicky Van Der Vekens, Fréderic De Rydt, Silvie Allaert, Marc Van De Velde, Jan Mulier

**Affiliations:** 1 Department of Anesthesiology, Reanimation and Intensive Care, AZ Sint Jan Brugge-Oostende, Brugge, Belgium; 2 Department of Anesthesia, Ghent University, Gent, Belgium; 3 Department of Anesthesia and Critical Care Medicine, Maria Middelares Hospital, Gent, Belgium; 4 Department of Cardiovascular Sciences, KU Leuven, Leuven, Belgium; 5 Department of Anesthesiology, UZ Leuven, Leuven, Belgium; 6 Department of Anesthesiology, KU Leuven - University of Leuven, Leuven, Belgium

## Correction to: Journal of Clinical Monitoring and Computing 10.1007/s10877-021-00803-z

The article: “Minimizing sevoflurane wastage by sensible use of automated gas control technology in the flow‑i workstation: an economic and ecological assessment”, written by Alain F. Kalmar, Nicky Van Der Vekens, Fréderic De Rydt, Silvie Allaert, Marc Van De Velde and Jan Mulier was originally published electronically on the publisher’s internet portal on 3 January 2022 without open access. With the author(s)’ decision to opt for Open Choice the copyright of the article changed on 19 February 2022 to ©The Author(s) 2022 and the article is forthwith distributed under a Creative Commons Attribution 4.0 International License, which permits use, sharing, adaptation, distribution and reproduction in any medium or format, as long as you give appropriate credit to the original author(s) and the source, provide a link to the Creative Commons licence, and indicate if changes were made. The images or other third party material in this article are included in the article’s Creative Commons licence, unless indicated otherwise in a credit line to the material. If material is not included in the article’s Creative Commons licence and your intended use is not permitted by statutory regulation or exceeds the permitted use, you will need to obtain permission directly from the copyright holder. To view a copy of this licence, visit http://creativecommons.org/licenses/by/4.0/. The original article has been corrected.

